# Considerations in Reference to Healthy in Contra-Distinction to Diseased Joints

**Published:** 1862-07

**Authors:** John Swineburne

**Affiliations:** Albany, New York


### CONSIDERATIONS IN REFERENCE TO HEALTHY, IN CONTRA-DISTINCTION TO DISEASED JOINTS.

From a Paper by JOHN SWINBURNE, M. D., Albany, New York.

In the Philadelphia *Medical and Surgical Reporter*, Vol. VII, p. 40, will be found an article, quoted from the San Francisco *Medical Press*, for July, in which Dr. E. S. Cooper lays down what he calls new surgical principles, and enumerates them from 1 to 7. In the main, I think the Doctor correct, but while all this may be true of a diseased joint, I would make the pertinent inquiry:

1st. Were you, as surgeon, to find a healthy knee joint, opened for two, three, or four inches in length, with a clean cutting instrument, would you allow it to heal by granulation? or would you rather close it by metalic sutures, as carefully as possible, with the object of obtaining union by the first intention?

2d. Were you called to treat a punctured wound of the same joint, would you close it, allow it to suppurate, or would you rather convert it into a full and free incision?

3d. In the operation for the removal of false cartilages from the joint, should your incision be large and free, and allow the same to heal by granulation? or should you strive to remove the cartilage by subcutaneous incision, and so heal the wound by the first intention?

There are some important considerations in reference to healty, in contra-distinction to diseased joints, which require further consideration, and I candidly confess (with my limited experience) that I am unable to give a solution of them. I would, therefore, invite the profession to investigate, carefully, this important and intricate matter.—*Philadelphia Med. and Surg. Reporter*.

[*To 1st Enquiry*—We would answer, that the parts should be brought together, with the view of effecting healing by first intention; but, at the same time, if the least purulent matter should be found in the joint afterwards, we would re-open it at once, and then make the wound heal by granulation.

*To 2d Enquiry.*—We would not convert the small punctured wound into a free incised one, immediately upon being called to such a case, if of recent occurrence, but would do so at once, afterwards, if we discovered that there existed a single drop of purulent matter in the joint.

*To 3d Enquiry.*—In removing foreign bodies from the knee joint, we always make an incision sufficiently free to remove the substance readily, and then, generally, favor healing of the wound by first intention, by bringing the parts together, but never resort to the subcutaneous section. We have, however, operated several times by direct incision, and afterwards applying lint in the wound caused it to heal by granulation, and in every case with favorable results; but were led to quit the plan in consequence of concluding that it kept the wounds somewhat longer in healing.

The rule we have fixed upon, after modifying our practice somewhat from that pursued when first we departed from the beaten track by disregarding the admission of atmosphere, is to favor the healing of fresh wounds of healthy joints by the adhesive process, but wounds of suppurating joints we make to heal by granulation.

REMARKS.—In the treatment of all wounds about the knee joint, whether accidental or made by the knife, we apply a roller around the limb as tightly as the patient can conveniently bear, commencing at the toes and continuing half way up the thigh. The pressure of the roller has a great influence in interrupting the mischievous process of suppuration and disorganization, which is the bane of these cases. A single drop of purulent matter is first generated from some portion of the injured synovial membrane, which, being of a soft and vascular structure, is disposed to suppurate under the influence of a slight inflammation; and this disposition to suppuration in a synovial membrane, together with the extent of development of this structure, in and around the joint, accounts for the rapidity with which purulent matter accumulates, in many cases after the slightest wounds, such as those made by the puncture of a penknife. The pressure of the roller interrupts, to a considerable extent, the flow of blood to this as well as all other soft structures entering into the formation of the joint.

Around the joint are numerous bursæ mucosæ, in which the disorganizing process often begins, though it may subsequently involve the entire knee joint. This is, by far, more frequently the case than any one not much accustomed to making incisions into suppurating cavities about the knee would form any idea of. We have repeatedly made incisions into what we supposed, prior to the operation, to be suppurating joint cavities, but found the purulent collection altogether external to the capsular ligament. In these cases, the same excrutiating pain and rapid accumulation of pus took place as occurs when the joint itself is previously involved. The application of a roller, immediately upon the reception of an injury involving the extra capsular tissues alone, would, in many instances, prevent the formation of purulent matter at all; but, when once it commences, nothing but opening the parts, freely, offers any prospect of an early cure.—*Editor San. Francisco Med. Press.*

